# Quantifying the recollection of discomfort and emotional suffering during a stay in intensive care: development and validation of the EXPRIM questionnaire: EXPRIM: quantification of discomfort experienced during an ICU stay

**DOI:** 10.1186/s41687-026-01011-6

**Published:** 2026-02-05

**Authors:** Pauline Minguet, Laura Gerard, Camille Colson, Sarah Neis-Gilson, Benoit Misset, Bernard Lambermont, Pierre Kalfon, J. Geoffrey Chase, Michael Beil, Olivier Bruyere, Charlotte Beaudart, Anne-Françoise Rousseau

**Affiliations:** 1 Intensive Care Department, University Hospital of Liège, University of Liège, Sart-Tilman B35, Avenue de l’Hôpital 1, Liège, B-4000 Belgium; 2 Vivalia, Hôpital de Libramont, Libramont, Belgium; 3 Réanimation polyvalente, Hôpital privé La Casamance, Aubagne, France; 4 Department of Mechanical Engineering, Centre for Bio-Engineering, University of Canterbury, Christchurch, New Zealand; 5 Clinical Pharmacology and Toxicology Research Unit, Department of Biomedical Sciences, Faculty of Medicine, Namur Research Institute for Life Sciences, University of Namur, Namur, Belgium; 6 Department of Medicine, National Health Service Highland, Inverness, UK; 7 Research Unit in Public Health, Epidemiology and Health Economics, University of Liège, Liège, Belgium; 8 Department of Sport and Rehabilitation Sciences, University of Liège, Liège, Belgium; 9 Research Unit for a Life-Course Perspective on Health & Education-RUCHE, University of Liège, Liège, Belgium

**Keywords:** Critical illness, Survivors, Discomfort, Suffering, Recollection

## Abstract

**Background:**

Discomfort perceived by patients during an intensive care unit (ICU) stay may limit their function, well-being, and quality of life after their stay. This study creates a comprehensive questionnaire (EXPRIM) able to distinguish and quantify the recollection of discomfort and emotional suffering experienced during a stay in an ICU stay, and to assess its performance.

**Method:**

The measurement properties of the questionnaire were tested in a first sample of 50 patients recruited between February and July 2024 in a tertiary ICU, using internal consistency, test-retest reliability, standard error of measurement (SEM), and construct validity. Content validity was also tested, in a second sample of 20 patients and 10 ICU healthcare providers recruited between April and June 2024 in a regional ICU, using face-to-face qualitative interviews based on a semi-structured interview guide assessing comprehensibility, relevance and comprehensiveness of the instruction and items.

**Results:**

EXPRIM is a 30-item questionnaire developed in French and translated in English. Its total score ranges from 0 to 10, reflecting the weighting of discomfort versus suffering. The internal consistency was very good (Cronbach’s alpha coefficient 0.81). The intra- and inter-examinator reliabilities were excellent (Intraclass Coefficient Correlation = 0.875 and 0.858, respectively). SEM ranged from −0.071 to 0.035. Good correlations were found between EXPRIM and other questionnaires measuring similar concepts. There was good evidence of content validity to explore recollection of the ICU stay.

**Conclusions:**

The EXPRIM questionnaire is a new tool with excellent psychometric properties to quantitatively assess the ICU experiences survivors recall after discharge. It is a promising tool to explore the extent of discomfort and emotional suffering during an ICU stay and to compare experiences between patients or populations.

**Supplementary Information:**

The online version contains supplementary material available at 10.1186/s41687-026-01011-6.

## Introduction

An intensive care unit (ICU) is a lifesaving and stressful environment [1]. Close monitoring and organ support generate a lot of stressors and traumatic events. Further, technological advancements that turn patients into a sum of parameters, poor working conditions, a lack of resources, or medical paternalism all contribute to dehumanization to varying degrees [2]. Twenty to thirty-five percent of ICU survivors have a poor or no memory of their stay in the intensive care unit [3, 4]. Some patients may report vivid recollection of their ICU stay, mentioning pleasant but also unpleasant memories. Pleasant memories seem to be mainly related to support and caring service [4]. Unpleasant memories are reported by 20% to 70% of patients, depending on the data source [4–7]. Physical sensations and difficult communication are two main affected domains [5]. These discomforts are expected to be more and more frequent as sedation practices are changing [8–10].

The discomfort perceived by ICU patients may be psychological and/or physical. They are related to the environment (known as environmental stressors), the specific ICU organization and the care provided [11]. They may be perceived as a simple unpleasant experience or may induce a real distress called suffering [12, 13]. In general, discomfort is mild, transient and non-invalidating. In contrast, emotional suffering brought on by discomforts affects patient’s functioning, well-being, and quality of life after the ICU stay [14]. Interestingly, a program aimed at reducing ICU discomfort was associated with a reduction of post-traumatic stress disorder (PTSD) prevalence one year after ICU discharge [15]. This result clearly suggests a link between ICU experience and psychological outcomes in ICU survivors. PTSD is part of the post-intensive care syndrome (PICS) and affects at least one of three ICU survivors [16], with a significant impact on health-related quality of life [17–19].

In most studies addressing the reported memories of an ICU stay [20], the recollection assessment was mainly based on unstructured interviews, or on qualitative tools [4, 5, 21]. Quantitative tools evaluating the unpleasant experience and suffering during an ICU stay are rare. The Edmonton Symptoms Assessment Scale (ESAS), developed to assess symptoms experienced by cancer patients [22], has been used in cancer patients receiving critical care [23]. Unfortunately, this tool has not been designed to assess specific ICU-related discomfort. The ICU Environmental Stressor Scale (ICUESS) is another questionnaire described more than 40 years ago [24]. Despite its validation in English, it has been rarely used in clinical studies [25, 26], likely due to its length and the items’ redundancy. Later, the “Inconforts des Patients de REAnimation” (IPREA) questionnaire was developed to evaluate discomfort during an ICU stay. It has been validated in French in two versions: a 16-item [27] and a 18-item questionnaire [28]. Its short format makes it acceptable for use in ICU survivors. This questionnaire’s shortcomings include the fact some types of discomfort are not investigated (such as relationships with healthcare providers, restraints, worries in link with social condition, … ), that there is no accurate differentiation between discomfort and emotional suffering, and that there is no overall score accounting for all the measured items (allowing only comparisons between the same common sources of ICU-related discomfort).

Clinical research and ICU management would benefit from a thorough questionnaire that distinguishes and quantifies the recall of discomfort and suffering experienced by patients during an ICU stay [29]. This study aims to create a tool meeting these requirements and, as a validation step, to characterise its performance.

## Design and methods

### Development of the new questionnaire

The questionnaire was developed in French and was denoted EXPRIM (« EXpérience Patient Réanimation: Inconforts et souffrance Morale »). The process was based on a three-stage validated method for the development of questionnaires aiming to measure patient reported outcome measures [30, 31]. Different experts in ICU care, post-ICU care and methodology were included in the development of this scale: an ICU physician former member of the Ethics Commission of the French Society of Intensive Care (B.M.), an ICU physician expert in post-ICU outcomes (AF. R.), three ICU nurses experts in post-ICU outcomes and follow-up (P.M., C.C., S.NG.), a clinical psychologist working in ICU (V.D.), and an expert in methodology of questionnaires (C.B.).

First, a comprehensive list of items about discomfort and emotional suffering recollected by patients during an ICU stay was drawn up from existing questionnaires intended to a similar population [24, 27, 28], from previously published studies about memories of ICU in critically ill patients [4, 6, 9, 32, 33] and from experiences of patients followed in our post-ICU clinic (their verbatim records are gathered in our dedicated database). This list included 20 themes related to environmental factors (noise, light, temperature), physical symptoms (pain, thirst, hunger), invasive procedures, emotional symptoms (anxiety, hallucinations), communication issues, restraints, autonomy, and informational concerns. This first list of 20 items was discussed with the experts in a meeting to reformulate some items, add or delete others, to ensure content relevance and conceptual saturation. Additions included: insufficient daylight, temporal/spatial landmarks, dyspnea, itching, boredom, difficulties expressing oneself, autonomy, physical activity limitations, and aspects related to interpersonal interactions with the ICU team (e.g., behaviour, communication). The most pertinent items were included in the questionnaire, increasing the list to 27 items. This list was submitted to some patients followed in our post-ICU clinic and to some ICU physicians and nurses during an intern symposium, where these ICU professionals were not part of the expert group and were not involved in the post-ICU follow-up. The principles of the Declaration of Helsinki were strictly applied by the investigator team during these interviews. Patients and ICU professionals gave oral feedback about this list during an unstructured interview. According to their comments, the list was then amended and corrected. Three additional items were added to address domains repeatedly reported as relevant but missing (insufficient information, inadequate or aggressive behaviour from the ICU team, concerns about social situation). This process resulted in the first complete 30-item version of the questionnaire. The entire progression—from the initial 20 items, to 27 items after expert expansion, to the 30-item first version of the questionnaire—is visually summarized in Supplemental Figure 1. All experts agreed on the relevance, and the necessity of the selected items.

**Fig. 1:**
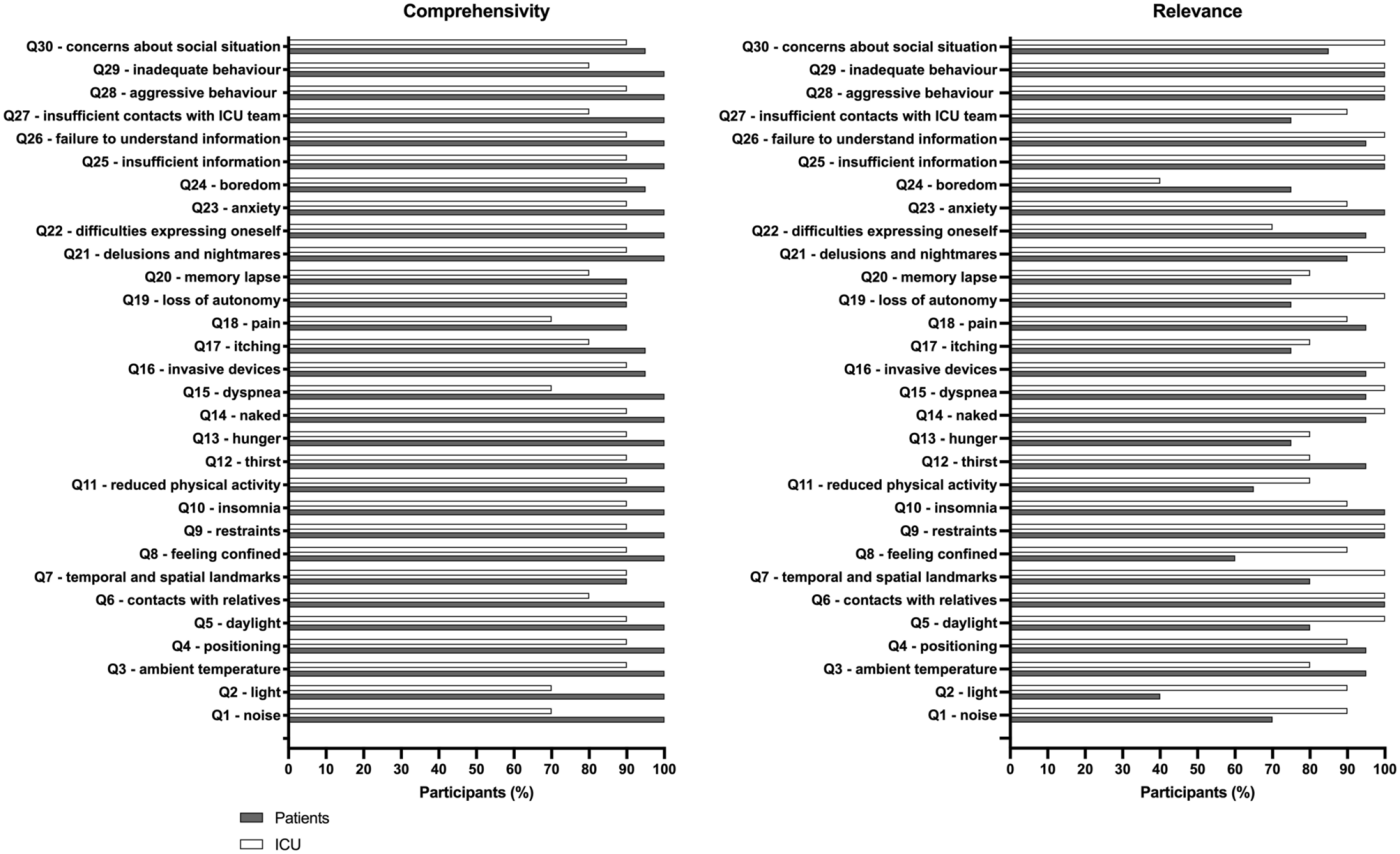
Proportion of participants (patients (*n* = 20) and experts *n* = 10)) reporting EXPRIM items as comprehensible or very comprehensible (**A**) and relevant or very relevant (**B**)

Second, an experts meeting was organized to define the layout of the questionnaire and the scoring system. Discussions focused on the classification of items, as well as the nuance between discomfort and emotional suffering and the weighting of the score needed to reflect this distinction. It has been decided to assign greater weight to emotional suffering, considering it a more impactful experience for the patient. The discussions resulted in the second version of the questionnaire, which was considered the as a pre-final French version.

Third, this pre-final version was submitted to a linguist (J.A.) to ensure it was free of any spelling or linguistic errors. The questionnaire was also submitted to 10 critically patients discharged from ICU for less than 7 days to ensure the understandability of each question and the acceptability of the questionnaire’s format. None of them suggested modifying the questionnaire. Following this pretest, the final version of the French EXPRIM questionnaire was established (Supplemental Figure 2).

The EXPRIM questionnaire is composed of 30 items. It generates two distinct subscores. First, the “E subscore” (“experience subscore”) quantifies the number of discomfort experienced during the ICU stay (range 0–30, as each reported discomfort contributes 1 point). Second, the “S subscore” (“emotional suffering subscore”) quantifies the number of experiences that patients identified as emotional suffering (range 0–120, with each such item contributing 4 points to reflect the greater clinical impact of suffering compared with discomfort alone). A global EXPRIM total score is then calculated to provide a standardized metric ranging from 0 to 10, using the following formula: [(number of positive E items + number of positive S items)/(30 + (number of positive E items*4))]*10. This scoring structure, established after multiple simulations, was designed to ensure that emotional suffering carries four times more weight than discomfort in the final score, while maintaining comparability across patients.

### Measurement properties of the EXPRIM scale

The psychometric validation study was performed considering the principles of the Consensus-based Standards for the Selection of Health Status Measurement Instruments (COSMIN) recommendations [34].

#### Ethics

Ethic approval of the study protocol was provided by the local ethics committee of our University Hospital on December 1^st^, 2023 (Local Ref: 2023/320).

#### Participants

A convenience sample of former ICU patients was recruited in our post-intensive care follow-up clinic (University Hospital of Liège, Belgium), during the first 7 days following ICU discharge. Exclusion criteria were a documented history of dementia, deafness or hearing loss, speech disorders and refusal to participate in the present research project. Patients were also excluded if they are unable to communicate in French, the local language. All enrolled patients were further screened for delirium, using the Confusion Assessment Method-ICU (CAM-ICU) instrument [35]. This four-feature standardized and validated tool enables non-psychiatrically trained clinicians to identify and recognize confusion and delirium quickly in both clinical and research settings. Patients detected with confusion or delirium (i.e., presence of features 1 and 2 and either 3 or 4) were further excluded from the validation study. Oral informed consent was obtained before enrolment by a member of the investigation team, not directly involved in patient’s clinical care. The instructions and the definition of the word “emotional suffering” were explained to the patients before the administration of the questionnaire.

#### Internal consistency

Internal consistency is defined as the degree of homogeneity across items [34] and is measured with the Cronbach’s alpha coefficient. This coefficient ranges from 0 to 1 with higher values representative of higher internal consistency. A value between 0.7 and 0.95 indicates a high level of internal consistency without significant risk of redundancy of items [34, 36]. To measure internal consistency, we first measured a global alpha coefficient for the EXPRIM questionnaire. We also assessed the impact of deleting each item on the total internal consistency.

#### Test–retest reliability

Test–retest reliability shows the extent to which the questionnaire produces the same scores for repeated measurements in subjects whose health status or memories have not changed. For this assessment, all patients completed the questionnaire 3 times. Because delirium is a fluctuating condition which could invalidate assessment, CAM-ICU screening was performed immediately before each administration. Patients with any positive CAM-ICU features were excluded from the validation process. To measure inter-examiner reliability, patients were asked to answer the questionnaire for a second time on the same day, approximatively 6 hours following the first administration. This short interval was chosen to allow a second assessment on the same day while leaving sufficient time to reduce short-term memory recall bias. This second interview was performed by a different clinical researcher (P.M., C.C., S.NG. or AF. R.) than the one who performed the first administration (L.G.). To measure intra-examiner reliability, the questionnaire was administered by the same clinical researcher (L.G.) a third time on the day following the first administration. This interval ensured the questionnaire was administered by the same investigator after a clinically relevant delay, while limiting the risk of significant changes in recollection. These short delays were intended to limit the risk of changes in recollection. Test–retest reliability was evaluated with the intraclass coefficient correlation (ICC) and its 95% confidence interval (95% CI). We used a two-way mixed method for absolute agreement. The reliability is considered as acceptable with an ICC of 0.7 [37].

The standard error of measurement (SEM) of the EXPRIM was also calculated. The SEM provides a range around the observed value in which the theoretical true value can be found. The SEM was calculated by dividing the standard deviation of the difference between the test and the retest by the square root of 2.

#### Construct validity

The evaluation of the construct validity of a questionnaire provides information on whether the questionnaire truly measures what it is supposed to measure. To assess convergent validity, correlations between EXPRIM and other questionnaires measuring similar concepts. Patients thus also completed two other assessment tools:

- the IPREA questionnaire (18-item version), which is a questionnaire with satisfactory psychometric properties, reporting the self-perceived ICU discomfort [28]. Patients are usually asked to rate the severity of each discomfort source experienced during the entire ICU stay on a 100-mm visual analog scale (VAS) displayed on a plastic sheet (0-no discomfort, 100-worst discomfort ever perceived). An overall assessment of discomfort is scored from 0 (minimal discomfort) to 100 (maximal discomfort). However, this scoring system did not allow a relevant comparison between the IPREA and the EXPRIM questionnaires: the IPREA score is calculated as a mean severity value, which tends to smooth or dilute individual discomfort experiences. For the purpose of assessing construct validity, we needed a scoring method that did not average out the results. As a total IPREA score was needed for comparison with the EXPRIM score, a binary transformation of IPREA responses was required. While this approach partially modifies the original score, it remains conceptually coherent: each IPREA item already has an embedded binary structure (0 = no discomfort; > 0 = discomfort), with an additional intensity rating layered on top. The scoring of the IPREA questionnaire was thus modified for the purposes of this study: one point was awarded for each item experienced by the patient. A total score thus ranged from 0 to 18.
- three different VAS quantifying the global ICU experience, the perceived discomfort and emotional suffering during the ICU stay, each rated from 0 to 10 (with 0 evoking a perfect experience and 10 the worst possible experience).

We made prior hypotheses and assumed that significant moderate to strong correlations would be observed between the EXPRIM (total or subscores), IPREA score (18-point score) and VAS. The following 8 hypothesis were formulated: we expected a moderate or strong correlation between EXPRIM total score and IPREA total score, between EXPRIM total score and VAS of global ICU experience, between the EXPRIM subscore E and VAS of unpleasant experiences, between EXPRIM subscore S and VAS of emotional suffering, between each EXPRIM subscores and IPREA total score, between the item 10 of EXPRIM and the item 4 of IPREA (assessing the discomfort or emotional suffering related to lack of sleep), and between the item 25 of EXPRIM and the item 16 of IPREA (related to the experience of receiving insufficient information about clinical status, treatment or plans). The construct validity was considered good if at least 75% of our hypotheses were confirmed by analyses [38].

#### Floor and ceiling effects

Floor and ceiling effects were considered to be present when more than 15% of the population obtained a maximum score (ceiling effect) or a minimum score (floor effect) [38]. When floor or ceiling effects are present, participants with the minimum or maximum score cannot be distinguished from one another, reducing the discriminative power of the questionnaire.

### Assessment of the content validity of the EXPRIM questionnaire

The content validity of the EXPRIM questionnaire was assessed following the COSMIN standard for evaluating the content validity of a patient-reported outcome measure [39], by conducting qualitative interviews with healthcare providers and patients. These interviews were performed by a clinical researcher of our team (L.G.). In line with the COSMIN methodology, we set a sample size of 20 patients and 10 healthcare providers as the target sample, recruited in another Belgian hospital (Vivalia, Hôpital de Libramont, Libramont, Belgium). To be included, patients had to be recently discharged from this hospital’s ICU, and healthcare providers had to work as physicians or nurses in this same ICU. Face-to-face interviews were conducted in French by a clinical researcher of our team (L.G.). A semi-structured interview guide was developed based on the COSMIN guidelines. All interviews began with a question measuring comprehensibility of the EXPRIM general instruction: patients answered by a yes/no answer, and healthcare providers were asked to rate their comprehensibility on a scale ranging from 1-not comprehensible to 4-very comprehensible. After that, EXPRIM was thoroughly reviewed item by item to assess the relevance (scale ranging from 1-not relevant at all to 4-very relevant) and the comprehensibility (scale ranging from 1-not comprehensible to 4-very comprehensible). Participants were also asked to comment on the response format of the questionnaire. Finally, participants where asked if they thought any items should be deleted from the questionnaire or if any item could be missing to offer a comprehensive assessment of any unpleasant experience related to their ICU stay.

### English translation

The translation process followed a five-stage validated template [40]. The EXPRIM questionnaire was first translated from French to English by two independent English speakers (J.G. C. and M.B.), both fluent in French. The two translators then synthesized the results of their two translations and agreed on a first consensual translated version of the questionnaire. Next, in stage three, two other independent bilingual translators with French as their mother tongue and fluent in English. (O.B. and J.P.), blinded to the original version French version of EXPRIM, independently translated the first English version back into French. In stage four, a committee composed of the four translators, a linguist and the research team members consolidated all of the versions of the questionnaire. The committee reviewed all of the translations and reached a consensus on any discrepancy. They also ensured equivalence between the source and target version in four areas: semantic, idiomatic, experiential and conceptual equivalences. They finally developed what would be considered the as a pre-final English version. Finally, the pre-final version was submitted as a pretest to a sample of 5 English-speaking ICU patients and nurses to assess its comprehensibility and clarity. Following this last step and taking into account potential last changes to increase understandability of the questionnaire, the English version of the EXPRIM was considered as the final one.

### Statistical analysis

A sample size power calculation was performed for reliability assessment since this is one of the most frequently used measurement properties. The “Wan Nor Arifin” web tool was used to calculate the sample size (https://wnarifin.github.io/ssc/ssicc.html). For an alpha of 0.01, a statistical power of 0.9, a minimum acceptable reliability of ICC = 0.7 and an expected ICC of 0.9, a total of 42 patients was required.

All analyses were carried out with the SPSS Statistics for MAC, version 29 (IBM Corporation, Armonk, NY, USA). The normality of the variables was checked by examining the histogram, the quantile-quantile plot, the Shapiro-Wilk test, and the difference between the mean and the median values. As most datasets did not pass the normality test, results were expressed as medians with interquartile ranges [P25 and P75] for quantitative parameters. Categorical variables were described by absolute and relative (%) frequencies. Comparisons between scores were assessed using the Wilcoxon matched-pairs ranked test. The correlation between two quantitative variables was assessed using the Spearman coefficient (r_s_). Difference between two qualitative variables was assessed using the Fisher’s exact test. The results were considered statistically significant at the 5% critical level.

## Results

### Development of the EXPRIM questionnaire

The pretest revealed no issues with the pre-final version of the questionnaire. The final French EXPRIM questionnaire is available in Supplemental Figure 2.

### Measurement properties of the EXPRIM questionnaire

#### Population

A total of 50 patients were recruited between February and July 2024. Descriptive characteristics of the included subjects are detailed in Table 1. The first administration of the questionnaires took place 5 [2–7] days after ICU discharge. Scores for the EXPRIM and the reference questionnaires are detailed in Table 2. It took 7 [5.4–9.4] minutes for the clinician to administer the questionnaire.

**Table 1:** Patients characteristics (ICU survivors recruited to measure the psychometric properties of the EXPRIM questionnaire)

Demographics		n = 50
Age, y		65 [50.7–71]
Male, n (%)		29 (58)
Admission type, n (%)	Medical	36 (72)
	Surgical	14 (28)
Organ failure at admission, n (%)	Cardiovascular	22 (44)
	Pulmonary	14 (28)
	Neurologic	5 (10)
	Other	9 (18)
ICU LOS, days		11.5 [9–16]

ICU: intensive care unit, LOS: length of stay

**Table 2:** Scores for the EXPRIM and reference questionnaires

Questionnaire (and range of possible score)		Observed score median [P25 and P75]	Observed minimum and maximum
EXPRIM	Total score (0–10)	5.8 [4.2–7.2]	0.9–8.8
	E subscore (0–30)	14 [9–16.25]	4–27
	S subscore (0–120)	36 [19-52]	0–92
IPREA (0–18)		7 [3.75–9]	0–15
VAS global experience (0–100)		3 [1–5.12]	0–10
VAS perceived discomforts (0–100)		4.5 [3–7.12]	0–10
VAS emotional suffering (0–100)		5 [2–8]	0–10

EXPRIM: EXpérience Patient Réanimation: Inconforts et souffrance Morale; IPREA: Inconforts des Patients de REAnimation; VAS: visual analogic scale; E subscore (experience subscore): items for which a discomfort was reported; S subscore (emotional suffering subscore): items for which the experience was described as emotional suffering

### Internal consistency

The Cronbach’s alpha for the entire questionnaire was 0.814, revealing a very good internal consistency. Deleting items did not modify the internal consistency: the lowest alpha was found when deleting the question 16 related to invasive devices (α = 0.797) and the highest alpha when deleting question 18 related to pain (α = 0.826) (Table 3).

**Table 3:** Results of internal consistency

EXPRIM questions and themes	Internal consistency Cronbach’s alpha if item deleted
Q1 - noise	0.799
Q2 – light	0.806
Q3 - ambient temperature	0.813
Q4 – positioning	0.807
Q5 – daylight	0.812
Q6 - contacts with relatives	0.804
Q7 - temporal and spatial landmarks	0.811
Q8 – feeling confined	0.804
Q9 – restraints	0.805
Q10 – insomnia	0.799
Q11 - reduced physical activity	0.813
Q12 – thirst	0.813
Q13 – hunger	0.809
Q14 – naked	0.815
Q15 – dyspnea	0.815
Q16 - invasive devices	0.797
Q17 – itching	0.814
Q18 – pain	0.826
Q19 - loss of autonomy	0.817
Q20 – memory lapse	0.800
Q21 - delusions and nightmares	0.812
Q22 - difficulties expressing oneself	0.799
Q23 – anxiety	0.803
Q24 – boredom	0.809
Q25 - insufficient information about clinical situation	0.805
Q26 - failure to understand information about clinical situation	0.813
Q27 - insufficient contacts with ICU team	0.808
Q28 - aggressive behaviour from ICU team	0.812
Q29 - inadequate behaviour from ICU team	0.813
Q30 - concerns about social situation	0.813

A Cronbach’s alpha between 0.7 and 0.95 indicates a high level of internal consistency without significant risk of redundancy of items

### Test–retest reliability

Total scores of EXPRIM were very similar between test and retest with the same examinator, respectively 5.8 [4.2–7.2] and 6.1 [3.9–7.7] (*p* = 0.787). Total scores of EXPRIM were also very similar between test and retest with two examinators, respectively 5.8 [4.2–7.2] and 6.05 [4.1–7.3] (*p* = 0.443). The intra-examiner and the inter-examiner reliability were excellent (Table 4). The SEM was calculated as 0.035 points, when considering difference between test and retest with a different examiner, and −0.071 points, when considering difference between test and retest with the same examiner.

**Table 4:** Results of reliability

EXPRIM	Intra-examiner reliability		Inter-examiner reliability	
	ICC	95% CI	ICC	95% CI
Subscore E	0.942	0.900 to 0.967	0.907	0.842 to 0.946
Subscore S	0.897	0.825 to 0.940	0.886	0.808 to 0.934
Total	0.875	0.790 to 0.927	0.858	0.763 to 0.917

CI: confidence interval; ICC: interclass correlation; E subscore (experience subscore): items for which a discomfort was reported; S subscore (emotional suffering subscore): items for which the experience was described as emotional suffering

### Construct validity

We validated 75% (6/8) of our hypothesis on convergent validity. Strong significant correlations were found between EXPRIM questionnaire total score and subscores and IPREA questionnaire. A moderate significant correlation was found between the EXPRIM questionnaire and the VAS assessing global ICU experience. Furthermore, no significant differences in proportions of patients who answered yes to question 10 in EXPRIM and question 4 in IPREA, as well as to question 25 in EXPRIM and question 16 in IPREA. However, weak correlations were found between EXPRIM subscores and the respective VAS (Table 5).

**Table 5:** Results of the convergent validity measurement

Hypothesis	**r**_**s**_	p value	Hypothesis validated?
EXPRIM total score vs IPREA: significant moderate to strong positive correlation	0.788	< 0.001	yes
EXPRIM total score vs VAS global experience: significant moderate to strong positive correlation	0.593	< 0.001	yes
EXPRIM subscore E vs IPREA: significant moderate to strong positive correlation	0.802	< 0.001	yes
EXPRIM subscore E vs VAS unpleasant experience: significant moderate to strong positive correlation	0.453	0.001	no
EXPRIM subscore S vs IPREA: significant moderate to strong positive correlation	0.832	< 0.001	yes
EXPRIM subscore S vs VAS emotional suffering: significant moderate to strong positive correlation	0.483	0.001	no
EXPRIM item 10 vs IPREA item 4: no significant difference	/	0.835	yes
EXPRIM item 25 vs IPREA item 16: no significant difference	/	0.659	yes

Item 10 of EXPRIM and item 4 of IPREA assess suffering related to lack of sleep. Item 25 of EXPRIM and item 16 of IPREA are related to the experience of receiving insufficient information about clinical status, treatment or plans

### Floor and ceiling effects

No patients obtained a score of 0 or the maximum score on the EXPRIM questionnaire. Therefore, neither floor nor ceiling effects were observed.

## Assessment of the content validity of the EXPRIM questionnaire

### Participants

A total of 30 interviews (20 with patients and 10 with ICU healthcare providers) were conducted between April and June 2024. Patients had a median age of 68 [54.2–72] years and recently spent 7.5 [4–11.7] days in ICU. Healthcare providers panel was composed of 3 ICU physicians, 1 head ICU nurse, 4 ICU nurses, 1 ICU physiotherapist and 1 ICU psychologist. ICU healthcare providers were 39 [32.7–44.7] years and a mean experience of 9.5 [5–20] years in intensive care.

### Comprehensibility

All patients expressed a clear understanding of the EXPRIM questionnaire instructions, including the need to distinguish the difference between experience of discomfort and suffering. Nine ICU healthcare providers (90%) reported instructions were sufficiently clear and appropriate. Comprehensibility of the different questions was good, higher in patients than in healthcare providers (Fig. 1). Questions 7 (about temporal and spatial landmarks) and 18 (about pain) were the most criticized, but no participants suggested the items should be rewritten. The answer format (yes/no) was considered as appropriate by 22/30 (73.3%) participants. Based on these comments, the questionnaire was left unchanged.

### Relevance

Overall, 95 [75–96.2]% of the patients and 90 [80–100]% of the ICU healthcare providers considered the items as relevant or very relevant. The less relevant questions for patients were related to noise or light experience, confinement feeling and reduced physical activities during the ICU stay (respectively Q1, Q2, Q8, Q11). Healthcare providers described only one question as less relevant, the one investigating boredom during the ICU stay (Q24) (Fig. 1). Since their relevance has been confirmed by experts and literature, these questions have been retained in the questionnaire.

### Comprehensiveness

Participants were asked if any question could be of limited relevance: 1/20 patients highlighted Q2 (about light) and Q30 (about social concerns) as not useful, and 3/10 healthcare providers highlighted Q17 (about itching) and Q24 (about boredom) as not useful. However, we were compelled to include these questions in the final version of the questionnaire due to the prevalence of these themes in the literature regarding perceived discomfort in the intensive care unit and, based on our experience, in the testimonies of former ICU patients. No patients thought some domains were missing. Some themes such as having trouble falling asleep, waiting too long for the nurse to come at bedside when called for help, feeling unheard, were pointed out as missing questions by 4/10 healthcare providers. However, we considered these topics were already covered by other questions, respectively Q10 (about insomnia), Q19 (loss of autonomy) and Q22 (difficulties expressing oneself) or Q27 (insufficient contacts with ICU team). Altogether, the questionnaire was finally kept unmodified.

### English translation

The instructions and the 30 items of the EXPRIM questionnaire were translated without any difficulties. The pretest revealed an issue with the understanding of the second item of the pre-final English version. Its translation has thus been changed according to the raised comment. The final English version of the EXPRIM questionnaire is available in the Supplemental Figure 3.

## Discussion

The present study allowed the development and the validation of the French EXPRIM tool, a 30-item questionnaire which provides a thorough and quantitative evaluation of ICU survivors’ recollections of discomfort and emotional suffering. Good measurement properties were observed during the validation process in former ICU patients. Internal consistency was considered excellent. Deleting items resulted did not change significantly the internal consistency, supporting the retention of all items of the EXPRIM questionnaire. No floor or ceiling effects were detected. Inter-examiner reliability was excellent, meaning results do not depend on the examiner who administer the questionnaire. Very low SEM values were observed, meaning we can be very confident that the “true” score of a patient can be found very close to the observed score. The convergent validity was confirmed with 75% of our hypothesis confirmed. We did not confirm a correlation between EXPRIM E and S subscores (respectively E subscore (experience subscore) corresponding to items for which a discomfort was reported, and S subscore (emotional suffering subscore) corresponding to items for which the experience was described as emotional suffering) and VAS assessing respectively unpleasant experiences or emotional suffering during the ICU stay. The weak correlations observed between the VAS and the EXPRIM subscores can be explained by several factors. First, VAS are simple, unidimensional instruments capturing a global subjective impression, whereas the EXPRIM questionnaire explores discomfort and suffering through 30 concrete, clearly defined items. This multidimensional and structured approach likely provides a more precise and nuanced assessment than a single overall rating. Second, prior to completing EXPRIM, patients received explicit definitions of key concepts such as ‘discomfort’ and ‘emotional suffering,’ which improves conceptual clarity. In contrast, the VAS items are broad and less context-specific, potentially leading to variability in how patients interpret and anchor their responses. Finally, asking patients to summarize a heterogeneous set of ICU experiences into a single numerical value may be challenging, especially in the early post-ICU period. This difficulty may contribute to the weaker-than-expected correlations with EXPRIM subscores, despite the conceptual proximity of the constructs assessed.

The comprehensibility and the comprehensiveness of the questionnaire, assessed by another panel of former ICU patients and ICU healthcare providers, were very good. The EXPRIM questionnaire explores indeed many of the common sources of discomfort during the ICU stay, as acknowledged by both patients and experts. Its administration by an ICU clinician (nurse, physician, psychologist) is a great chance to talk with the patient and to try to figure out why they experienced discomfort or suffering. However, clinician-administered questionnaires also have disadvantages. They may introduce a social desirability bias, potentially leading patients to underreport discomfort or suffering. This approach also requires staff time. In addition, the interaction may vary depending on the interviewer’s communication style, which could introduce variability in the responses. Despite this bias, the feedback provided by the questionnaire is precious for the ICU team. It raises awareness about the experience of a stay in ICU, it questions practices and allows for a subsequent continuous quality of care improvement [41, 42]. For this reason, we advise against using self-administration of the EXPRIM questionnaire.

EXPRIM is intended to be administered once, after ICU discharge, as a photo of patient’s recollection. Thus, there was no calculation of the smallest detectable change and no evaluation of the responsiveness and sensitivity to change. We suggest administering the EXPRIM questionnaire during the few days following ICU discharge. For relevance, we suggest completing the questionnaire after making sure there are no significant cognitive disorders present, which is a common prerequisite for any form of assessment of the clinical status of a fragile patient recently discharged from ICU. An earlier administration, before ICU discharge, may be considered. However, patients may not be available for such an interview on the day of ICU discharge if they have ongoing cognitive impairments or if they anticipate issues with the transfer to the ward. An administration earlier during ICU stay could allow a prompt detection of patient’s discomfort and emotional suffering, thus favouring the implementation of corrective interventions. This timeframe has not been retained and investigated in the present validation study. The IPREA questionnaire has been used for this purpose in some studies [42, 43]. However, the IPREA, as well as the EXPRIM may be too complex in view of the frequent impaired cognitive capacities observed in critically ill patients. Further, a global score is probably not needed at this stage of the critical illness trajectory, simple questions about how the patient feels can be enough if the aim is to improve the ongoing ICU stay related experience. Overall, there are benefits and drawbacks to both memory-based (after the intensive care unit stay) and moment-based (in the ICU) evaluation of perceived discomfort, depending on the assessment’s objective and the prevalence and duration of cognitive disorders. It would be pertinent to compare the two scores in future research. Given completing the questionnaire took less than ten minutes, EXPRIM administration is feasible. The EXPRIM questionnaire appears to take less time to complete than the IPREA questionnaire, likely because EXPRIM was administered to patients who have regained their cognitive abilities and that it does not ask for a numerical estimate of the severity of each discomfort.

The special marking system of the EXPRIM tool allows the quantification of the perceived discomfort, which is further modulated by the importance of reported suffering. Indeed, experiencing discomfort is not always synonymous with emotional suffering: the feeling and the meaning of the experience is unique to each patient. In addition, there is no possibility of suffering in the absence of unpleasant experience. The maximum theoretical score is thus different for every single patient. Ultimately, the rating is converted to a 10-point total score to enable comparisons between patients while accounting for the severity of their suffering. This marking system makes EXPRIM particularly relevant to investigate the relationship between the ICU stay experience and the long-term psychological outcomes, especially PTSD. The possible interest of the EXPRIM score as a post-ICU PTSD predictor should also be further investigated. In addition, using this total score could be useful for the ICU teams to better understand how to improve the ICU environment and to further assess the efficiency of strategies aiming at reducing ICU-related suffering. The distinction between discomfort and emotional suffering could help identifying actions which have a high priority to reduce patients suffering, as some strategies may have an impact on emotional suffering while not eliminating discomfort. Such outcomes are offered by relaxation and hypnosis, for example. This research topic is essential, since the impact of the various degree of discomforts and suffering on post-ICU outcomes is still poorly studied.

## Strengths and limitations

This study has specific strengths. First, we respected guidelines and followed rigorous steps, which have been previously used with success in many other studies. Second, a sufficient sample size was recruited to evaluate the psychometrics of the questionnaire.

However, this study has also some limitations. First, the sample size was calculated for reliability assessment of the measurement properties. However, this sample size was also appropriate to assess the other measurement properties, in accordance with the recommendations on this topic [38]. Second, we did not conduct a systematic review to compile an exhaustive list of all the discomforts that patients encountered while in the intensive care unit. However, EXPRIM incorporates the discomforts ICU survivors most frequently report, based on our extensive experience with post-ICU follow-up, and based on the different concordant published studies. Third, the scoring of the comparator questionnaire (IPREA) was partially modified for this study, in the absence of alternative validated tools available for comparison. Although this adaptation should be acknowledged as a limitation, one of the two fundamental components of the original IPREA assessment—whether a discomfort was present or absent—was still fully preserved despite this modification. Fourth, retests were scheduled in the 24 hours following the test, aiming to limit the risk of altered memories. In contrast, it could have induced a recall bias, as participants may have still remembered the answers, they gave hours or the day before. However, we assumed the number of items could have limited this risk. Finally, this study did not produce a cut-off score. There is most likely no cut-off score to establish, except zero, since the EXPRIM questionnaire explores feelings and experiences, that are completely subjective.

## Conclusion

We developed and validated the EXPRIM questionnaire to quantitatively assess the ICU experiences survivors recall after discharge, differentiating discomfort from emotional suffering. Its psychometrics properties are excellent, making it a possible relevant tool to compare perceived ICU experiences between patients or populations. In the next future, EXPRIM could help investigating the effectiveness of in-ICU humanization strategies on patients experience and secondarily on psychological outcomes.

## Electronic supplementary material

Below is the link to the electronic supplementary material.

Supplementary Material 1

## Data Availability

The datasets used and/or analyzed during the currentstudy are available from the corresponding author on reasonable request.

## Abbreviations

CAM-ICU: Confusion Assessment Method-ICU
ESAS: Edmonton Symptoms Assessment Scale
EXPRIM: EXpérience Patient Réanimation: Inconforts et souffrance Morale
ICC: Interclass coefficient correlation
ICU: Intensive care unit
ICUESS: ICU Environmental Stressor Scale
IPREA: Inconforts des Patients de REAnimation
LOS: Length of stay
PICS: Post-intensive care syndrome
PTSD: Post-traumatic stress disorders
SDC: Smallest detectable change
SEM: Standard error of measurement
VAS: Visual analogic scale
