# Supplementary material for: Quantifying the recollection of discomfort and emotional suffering during a stay in intensive care: development and validation of the EXPRIM questionnaire: EXPRIM: quantification of discomfort experienced during an ICU stay
Source: J Patient Rep Outcomes. 2026 Feb 5;10:38. doi: 10.1186/s41687-026-01011-6 (PMC12972440; doi:10.1186/s41687-026-01011-6)
Supplement: Supplementary file 1 — Supplementary Material 1 [file 41687_2026_1011_MOESM1_ESM.docx]

**Quantifying the recollection of discomfort and emotional suffering during a stay in intensive care: development and validation of the EXPRIM questionnaire.**

**Supplemental Material**

Supplemental Figure 1: Description of how the items that make up the EXPRIM questionnaire were selected by the experts group.

*Abbreviations: info: information; resp: respiratory*

**

Supplemental Figure 2: The EXPRIM questionnaire (French version)

**Questionnaire EXPRIM**

**Expérience Patient en Réanimation : Inconforts et souffrances Morales**

Consigne :

Ce questionnaire s’adresse aux patients ayant séjourné aux soins intensifs. Il a pour but de recenser les éventuels inconforts et/ou souffrances vécus lors de ce séjour.

Le questionnaire est administré de la manière suivante : « À propos des situations énoncées, les avez-vous vécues durant votre séjour aux soins intensifs ? ». Les réponses possibles sont « oui » ou « non ». Si la réponse est « oui », la question suivante est : « Était-ce une source de souffrance pour vous ? ». La réponse à cette seconde question est à nouveau « oui » ou « non ».

N.B. : Par souffrir/souffrance, on entend un état prolongé de détresse morale.

| **Items** | | **Items V** | | **Items S** | |
| --- | --- | --- | --- | --- | --- |
| **À propos des situations suivantes…** | | **Les avez-vous vécues ?** | | **Était-ce une souffrance ?** | |
|  |  | **Oui**  **= 1** | **Non**  **= 0** | **Oui**  **= 4** | **Non**  **= 0** |
| 1 | Entendre trop de bruit |  |  |  |  |
| 2 | Percevoir trop de lumière |  |  |  |  |
| 3 | Avoir trop chaud ou trop froid |  |  |  |  |
| 4 | Être dans une position inconfortable (au lit ou au fauteuil) |  |  |  |  |
| 5 | Manquer de la lumière du jour |  |  |  |  |
| 6 | Manquer de contacts avec mes proches |  |  |  |  |
| 7 | Manquer de repères spatio-temporels (lieu, date, heure, jour) |  |  |  |  |
| 8 | Avoir une sensation d’enfermement, ne pas pouvoir sortir de ma chambre |  |  |  |  |
| 9 | Être attaché ou empêché de bouger |  |  |  |  |
| 10 | Manquer de sommeil |  |  |  |  |
| 11 | Manquer d’activité physique |  |  |  |  |
| 12 | Avoir soif |  |  |  |  |
| 13 | Avoir faim |  |  |  |  |
| 14 | Être nu devant les soignants |  |  |  |  |
| 15 | Avoir des difficultés à respirer |  |  |  |  |
| 16 | Être gêné par des tuyaux ou le matériel de support de mes fonctions vitales |  |  |  |  |
| 17 | Avoir des démangeaisons |  |  |  |  |
| 18 | Avoir mal |  |  |  |  |
| 19 | Dépendre des soignants ou de mes proches |  |  |  |  |
| 20 | Avoir un « trou noir » relatif au séjour aux soins intensifs *(càd une période durant laquelle vous n’avez, à ce jour, pas de souvenirs)* |  |  |  |  |
| 21 | Avoir des hallucinations / faire des cauchemars |  |  |  |  |
| 22 | Avoir des difficultés pour m’exprimer et me faire comprendre |  |  |  |  |
| 23 | Ressentir de l’anxiété / de l’angoisse |  |  |  |  |
| 24 | M’ennuyer |  |  |  |  |
| 25 | Manquer d’informations concernant mon état de santé, l’organisation des soins et/ou les examens prévus |  |  |  |  |
| 26 | Ne pas comprendre l’information donnée par les soignants |  |  |  |  |
| 27 | Manquer de contacts avec le personnel soignant |  |  |  |  |
| 28 | Percevoir un comportement (qu’il soit verbal ou physique) **agressif** de la part du personnel soignant à mon égard |  |  |  |  |
| 29 | Percevoir un comportement (qu’il soit verbal ou physique) **inadapté** de la part du personnel soignant *(tutoiement, infantilisation, etc.)* à mon égard |  |  |  |  |
| 30 | Avoir des préoccupations en lien avec le déroulement de ma vie privée durant mon absence : ma famille, mon emploi, ma maison, mon argent, mon animal de compagnie, etc. |  |  |  |  |
|  | Sous-scores | V = ……… |  | S = ……… |  |
|  | Score total=  [(nombre items V+ nombre items S) /(30+(nombre item V*4))]*10 | = …… /10 | | | |

Supplemental Figure 3: The EXPRIM questionnaire (English version)

**EXPRIM Questionnaire**

**Patient Experience in Intensive Care: Discomfort and Emotional Suffering**

Instructions:

This questionnaire has been designed for patients who have been admitted to intensive care. Its purpose is to identify any potential discomfort and/or suffering experienced during your stay.

The questionnaire is administered in the following manner: “Regarding the situations described, did you experience them during your stay in intensive care? The possible responses for each are: “Yes” or “No”. If the response is “Yes”, the next question is: “Was this situation a source of emotional suffering for you?”. The possible responses to this second question are again: “Yes” or “No”.

Please note: By emotional suffering, we mean a prolonged state of mental distress.

| **Items** | | **E items** | | **S items** | |
| --- | --- | --- | --- | --- | --- |
| **Regarding the following situations…** | | **Did you experience this situation?** | | **Was this a source**  **of emotional suffering?** | |
|  |  | **Yes**  **= 1** | **No**  **= 0** | **Yes**  **= 4** | **No**  **= 0** |
| 1 | Hearing too much noise |  |  |  |  |
| 2 | Perceiving the environment as too bright |  |  |  |  |
| 3 | Feeling too hot or too cold |  |  |  |  |
| 4 | Being in an uncomfortable position (in bed or in a chair) |  |  |  |  |
| 5 | Lacking daylight |  |  |  |  |
| 6 | Lacking contact with my loved ones |  |  |  |  |
| 7 | Lacking spatial or temporal reference points (place, date, time, day) |  |  |  |  |
| 8 | Feeling trapped or being unable to leave my room |  |  |  |  |
| 9 | Being restrained or prevented from moving |  |  |  |  |
| 10 | Lacking sleep |  |  |  |  |
| 11 | Lacking physical activity |  |  |  |  |
| 12 | Feeling thirsty |  |  |  |  |
| 13 | Feeling hungry |  |  |  |  |
| 14 | Being naked in front of healthcare workers |  |  |  |  |
| 15 | Having difficulty breathing |  |  |  |  |
| 16 | Being bothered by tubes or life-support equipment |  |  |  |  |
| 17 | Experiencing itching |  |  |  |  |
| 18 | Experiencing pain |  |  |  |  |
| 19 | Feeling dependent on healthcare workers or my loved ones |  |  |  |  |
| 20 | Having a “blackout” regarding my intensive care stay (i.e. a period of which you currently have no memories) |  |  |  |  |
| 21 | Experiencing hallucinations and/or nightmares |  |  |  |  |
| 22 | Having difficulty expressing myself and/or making myself understood |  |  |  |  |
| 23 | Feeling anxious or distressed |  |  |  |  |
| 24 | Feeling bored |  |  |  |  |
| 25 | Lacking information about my health, the organization of my care, and/or the examinations planned |  |  |  |  |
| 26 | Not understanding the information provided by healthcare workers |  |  |  |  |
| 27 | Lacking contact with healthcare workers |  |  |  |  |
| 28 | Perceiving aggressive behavior (verbal or physical) towards me from healthcare workers |  |  |  |  |
| 29 | Perceiving inappropriate behavior (verbal or physical) towards me from healthcare workers (e.g. using informal address, patronizing language, etc.) |  |  |  |  |
| 30 | Having concerns related to my private life during my stay, such as my family, my job, my house, my money, my pets, etc.? |  |  |  |  |
|  | Sub-scores | E = ……… |  | S = ……… |  |
|  | Total score=  [(number of E items+ number of S items) /(30+(number of E item*4))]*10 | = …… /10 | | | |
